# Supplementary material for: Pluchea dioscoridis extract: A novel therapeutic approach for polycystic ovary syndrome targeting ovarian morphology, dopamine pathways, and neurobehavior in relation to its phytocomponents
Source: PLoS One. 2025 Jun 26;20(6):e0325439. doi: 10.1371/journal.pone.0325439 (PMC12200721; doi:10.1371/journal.pone.0325439)
Supplement: Attachment — Submitted filename: Response to Reviewers.docx [file pone.0325439.s002.docx]

**Rebuttal List**

Dear Editor:

In response to the revision of our manuscript entitled:

PONE-D-25-08188

Pluchea dioscoridis Extract: A Novel Therapeutic Approach for Polycystic Ovary Syndrome Targeting Ovarian Morphology, Dopamine Pathways, and Neurobehavior in Relation to its Phytocomponents

We would like to thank the editors and reviewers for their efforts in reviewing the manuscript and raising its quality. Herein are point-by-point author response for the editor and reviewers’ comments.

Journal requirements:

When submitting your revision, we need you to address these additional requirements.

1.  Please ensure that your manuscript meets PLOS ONE's style requirements, including those for file naming. The PLOS ONE style templates can be found at

[https://journals.plos.org/plosone/s/file?id=wjVg/PLOSOne_formatting_sample_main_body.pdf](https://track.editorialmanager.com/CL0/https:%2F%2Fjournals.plos.org%2Fplosone%2Fs%2Ffile%3Fid=wjVg%2FPLOSOne_formatting_sample_main_body.pdf/1/010f0195a7550721-abcf9d56-8ea8-4ab8-b2a9-dac657f9b08e-000000/0kkypjHTSBe6HXILM70UKp4KA2SU_1X488QV5F0LpDs=202)  and

[https://journals.plos.org/plosone/s/file?id=ba62/PLOSOne_formatting_sample_title_authors_affiliations.pdf](https://track.editorialmanager.com/CL0/https:%2F%2Fjournals.plos.org%2Fplosone%2Fs%2Ffile%3Fid=ba62%2FPLOSOne_formatting_sample_title_authors_affiliations.pdf/1/010f0195a7550721-abcf9d56-8ea8-4ab8-b2a9-dac657f9b08e-000000/GlhFDwnmzh6zybacN8wZAmVJYyyKkNli2vIOXDCg2cI=202)

Author response: the manuscript was modified to meet the PLOS ONE's style

2. When completing the data availability statement of the submission form, you indicated that you will make your data available on acceptance. We strongly recommend all authors decide on a data sharing plan before acceptance, as the process can be lengthy and hold up publication timelines. Please note that, though access restrictions are acceptable now, your entire data will need to be made freely accessible if your manuscript is accepted for publication. This policy applies to all data except where public deposition would breach compliance with the protocol approved by your research ethics board. If you are unable to adhere to our open data policy, please kindly revise your statement to explain your reasoning and we will seek the editor's input on an exemption. Please be assured that, once you have provided your new statement, the assessment of your exemption will not hold up the peer review process.

Author response: Noted with thanks

3. Please include a separate caption for each figure in your manuscript.

 Author response: A separate caption was added.

**Comments to the Author**

1. Is the manuscript technically sound, and do the data support the conclusions?

The manuscript must describe a technically sound piece of scientific research with data that supports the conclusions. Experiments must have been conducted rigorously, with appropriate controls, replication, and sample sizes. The conclusions must be drawn appropriately based on the data presented.

Reviewer #1: Yes

Reviewer #2: Yes

2. Has the statistical analysis been performed appropriately and rigorously?

Reviewer #1: Yes

Reviewer #2: Yes

3. Have the authors made all data underlying the findings in their manuscript fully available?

The [PLOS Data policy](http://track.editorialmanager.com/CL0/http:%2F%2Fwww.plosone.org%2Fstatic%2Fpolicies.action%23sharing/1/010f0195a7550721-abcf9d56-8ea8-4ab8-b2a9-dac657f9b08e-000000/CsljrVQzBw0ltgdYTgvwIOBXNtjFvuhY9oqMH7GQWOk=202) requires authors to make all data underlying the findings described in their manuscript fully available without restriction, with rare exception (please refer to the Data Availability Statement in the manuscript PDF file). The data should be provided as part of the manuscript or its supporting information, or deposited to a public repository. For example, in addition to summary statistics, the data points behind means, medians and variance measures should be available. If there are restrictions on publicly sharing data—e.g. participant privacy or use of data from a third party—those must be specified.

Reviewer #1: Yes

Reviewer #2: Yes

4. Is the manuscript presented in an intelligible fashion and written in standard English?
PLOS ONE does not copyedit accepted manuscripts, so the language in submitted articles must be clear, correct, and unambiguous. Any typographical or grammatical errors should be corrected at revision, so please note any specific errors here.

Reviewer #1: No

Reviewer #2: Yes

Author response: the manuscript was revised grammatically.

5. Review Comments to the Author

Please use the space provided to explain your answers to the questions above. You may also include additional comments for the author, including concerns about dual publication, research ethics, or publication ethics. (Please upload your review as an attachment if it exceeds 20,000 characters)

Reviewer #1:

**In the abstract, it is mentioned that: all the treatments were for 21 days. It seems that it should be** revised since after 21 days of letrozole for PCOS induction, metformin and P. dioscoridis was used.

Author response: Many thanks to the reviewer for this valuable comment. We revised this part in the abstract.
**The result section of the abstract must be written in a way to shows the significance or non-**significant of changes statistically.

Author response: We agree with the reviewer regarding this comment. We modified and highlighted this issue.
**In the Introduction add a reference for this sentence: Animal models of depression, including the** learned helplessness and chronic mild stress (CMS) models, demonstrate substantial alterations in the dopamine (DA) system.
**Also, add a reference for this: This encompasses modified dopamine receptor expression in** limbic areas, diminished synaptic dopamine release, lowered levels of the dopamine metabolite homovanillic acid, and decreased dopaminergic activity in the striatum.
**The below studies are suggested:**
The chronic mild stress (CMS) model of depression: History, evaluation and usage

Author response: We appreciate the reviewer’s comment. We added references to these sentences and were highlighted.

Added references:

P. Belujon and A. A. Grace, “Dopamine system dysregulation in major depressive disorders,” *Int J Neuropsychopharmacol*, vol. 20, no. 12, pp. 1036–1046, Dec. 2017, doi: 10.1093/ijnp/pyx056.

P. L. Reddy, S. Khanna, M. N. Subhash, S. M. Channabasavanna, and B. S. Rao, “CSF amine metabolites in depression,” *Biol. Psychiatry*, vol. 31, no. 2, pp. 112–118, Jan. 1992, doi: 10.1016/0006-3223(92)90198-9.

J. C. Pruessner, F. Champagne, M. J. Meaney, and A. Dagher, “Dopamine release in response to a psychological stress in humans and its relationship to early life maternal care: a positron emission tomography study using [11C]raclopride,” *J Neurosci*, vol. 24, no. 11, pp. 2825–2831, Mar. 2004, doi: 10.1523/JNEUROSCI.3422-03.2004.

P. Willner, “The chronic mild stress (CMS) model of depression: History, evaluation and usage,” *Neurobiol. Stress*, vol. 6, pp. 78–93, Feb. 2017, doi: 10.1016/j.ynstr.2016.08.002.

**Method:**
For this section add reference: The air-dried plant materials (820 gm) were macerated in 4 liters of a 7:3 mixture of ethanol and distilled water for 5 days at the room temperature then filtered. After completing three rounds of this process, all the liquor extracts were gathered and dried under vacuum, producing 34.2 g of black gum that was kept refrigerated at 4 °C until additional testing was done.
**The below study is suggested**:
Capacity of Mentha spicata (spearmint) Extract in Alleviating Hormonal and Folliculogenesis Disturbances in Polycystic Ovarian Syndrome Rat Model

Author response: The reference was inserted.
added reference:

M. J. Bagheri, M. S. Ataabadi, F. Koohpeyma, and S. Alaee, “Capacity of Mentha spicata (spearmint) Extract in Alleviating Hormonal and Folliculogenesis Disturbances in Polycystic Ovarian Syndrome Rat Model,” J. Worlds Poult. Res., vol. 10, no. 3, pp. 451–456, Sep. 2020, doi: 10.36380/scil.2020.wvj56.
 **Add the weight of rats.**

Author response: The weight was added and highlighted in the corresponding section.

**Add a reference for the selection of dose of letrozole**. In most of the studies 28 days of letrozole administration is used.

**dose selection using studies such as below:**
Blood volatile organic compounds as potential biomarkers for poly cystic ovarian syndrome (PCOS): An animal study in the PCOS rat model

Author response: thanks editor for this comment. the reference was added.

Added references:

Y.-H. Zheng, Y. Xu, H.-X. Ma, C.-J. Liang, and T. Yang, “Effect of High-Fat Diet on the Intestinal Flora in Letrozole-Induced Polycystic Ovary Syndrome Rats,” *Evid. Based Complement. Alternat. Med.*, vol. 2021, p. e6674965, Jun. 2021, doi: 10.1155/2021/6674965.

M. S. Ataabadi, S. Bahmanpour, S. Yousefinejad, and S. Alaee, “Blood volatile organic compounds as potential biomarkers for poly cystic ovarian syndrome (PCOS): An animal study in the PCOS rat model,” *J. Steroid Biochem. Mol. Biol.*, vol. 226, p. 106215, Feb. 2023, doi: 10.1016/j.jsbmb.2022.106215.

.

**It is suggested to explain about the dose selection using studies such as below:**
Blood volatile organic compounds as potential biomarkers for poly cystic ovarian syndrome (PCOS): An animal study in the PCOS rat model

Author response: Many thanks to the reviewer for this comment. The duration of letrazole-induced PCOS model was based on a pilot study and on a study done by Zheng et al., 2021

Supporting reference:

Y.-H. Zheng, Y. Xu, H.-X. Ma, C.-J. Liang, and T. Yang, “Effect of High-Fat Diet on the Intestinal Flora in Letrozole-Induced Polycystic Ovary Syndrome Rats,” *Evid. Based Complement. Alternat. Med.*, vol. 2021, p. e6674965, Jun. 2021, doi: 10.1155/2021/6674965.

**Also use other studies which used metformin or P. dioscoridis and explain about the criteria for selecting similar or different doses in comparison to other studies.**
For example, in the below study metformin 500 for 1 week was used instead of 300 for 21 days.
The effects of melatonin and metformin on histological characteristics of the ovary and uterus in letrozole-induced polycystic ovarian syndrome mice: A stereological study

Author response: we appreciate the reviewer for this comment. There were different doses according to the species, mode of administration, so the reference here was for mice and metformin was given intraperitoneal while our study, rats were given metformin intragastric. The dosage, duration, and mode of metformin administration was based on W. Liu, W. Liu, Y. Fu, Y. Wang, and Y. Zhang, “Bak Foong pills combined with metformin in the treatment of a polycystic ovarian syndrome rat model,” *Oncol. Lett.*, vol. 10, no. 3, pp. 1819–1825, Sep. 2015, doi: 10.3892/ol.2015.3466.In addition we tried to standardize the administration method and duration of treatment in metformin and ***P. dioscoridis.*** We added this explanation to the methodology section.

**Supporting references:**

W. Liu, W. Liu, Y. Fu, Y. Wang, and Y. Zhang, “Bak Foong pills combined with metformin in the treatment of a polycystic ovarian syndrome rat model,” *Oncol. Lett.*, vol. 10, no. 3, pp. 1819–1825, Sep. 2015, doi: 10.3892/ol.2015.3466.

A. I. Elshamy, A. E. N. El Gendy, A. R. Farrag, and M. Nassar, “Antidiabetic and antioxidant activities of phenolic extracts of Conyza dioscoridis L. shoots,” Int. J. Pharm. Pharm. Sci., vol. 7, pp. 65–72, Jan. 2015.

**In this section mention the temperature or method for preservation for subsequent studies: Both brain and ovaries were collected for further biochemical and histopathological studies.**

Author response: we appreciate the reviewer for this comment. Modification was added and highlighted in the methodology section.

-Both brain and right ovaries were collected and stored at -20 °C for further biochemical, while the left ovaries were fixed in Bouin fixative for histopathological studies.

**If the authors established the method for Ki 67 immunostaining, it is suggested to add its reference.**

Author response: The references were added.

Added references:

S. Furat Rencber *et al.*, “Effect of resveratrol and metformin on ovarian reserve and ultrastructure in PCOS: an experimental study,” *J. Ovarian Res.*, vol. 11, no. 1, p. 55, Jun. 2018, doi: 10.1186/s13048-018-0427-7.

S. E. Ali *et al.*, “Novel promising reproductive and metabolic effects of Cicer arietinum L. extract on letrozole induced polycystic ovary syndrome in rat model,” *J. Ethnopharmacol.*, vol. 278, p. 114318, Oct. 2021, doi: 10.1016/j.jep.2021.114318.

**If the primers are designed by the authors it is suggested to mention the software or if they were selected from the studies add reference.**

Author response: the references were added in table1 **Results
Delete repetitive numbers in the result section if they are presented in figures or tables**.

Author response: Thanks to the reviewer for this comment. Repetitive numbers were deleted.

**Reviewer #2:**

**Title** The title is comprehensive.
**Abstract**
**The abstract provides a clear overview of the study's objectives, methods, results, and conclusions.
The introduction is well structured.
The methods section is well-structured.**

Author response: we would like to thank the reviewer for his/her effort in reviewing the paper and raising its quality and for this encouraging comments.

**some figures (e.g., Figure 5) could be improved for clarity, particularly in labeling and the presentation of statistical significance.**

Author response: we improved the figures clarity.

**Additionally, the results section could benefit from a more detailed discussion of the chemical profile of P. dioscoridis and how specific phytocomponents may contribute to the observed effects.**

Author response: Thank you for your insightful suggestion. We agree that a more detailed discussion of the chemical profile of *P. dioscoridis* and its phytocomponents would enhance the interpretation of our findings. Accordingly, we have expanded the Results and Discussion sections to include a more comprehensive analysis of the known bioactive constituents of *P. dioscoridis*, such as flavonoids (e.g., luteolin), phenolic acids, and nitrogenous compounds. We also discussed how these compounds—based on existing literature—may underlie the observed pharmacological effects, supporting a possible mechanistic link. Added parts are highlighted in yellow in the corresponding sections.

Supporting references:

Elgamal, A. M., Ahmed, R. F., Abd-ElGawad, A. M., El Gendy, A. E. G., Elshamy, A. I., & Nassar, M. I. (2021). Chemical Profiles, Anticancer, and Anti-Aging Activities of Essential Oils of Pluchea dioscoridis (L.) DC. and Erigeron bonariensis L. Plants (Basel, Switzerland), 10(4), 667. https://doi.org/10.3390/plants10040667

Malarz, J., Michalska, K., & Stojakowska, A. (2024). Polyphenols of the Inuleae-Inulinae and Their Biological Activities: A Review. Molecules, 29(9), 2014. <https://doi.org/10.3390/molecules29092014>

Paparella, R., Panvino, F., Leonardi, L., Pucarelli, I., Menghi, M., Micangeli, G., Tarani, F., Niceta, M., Rasio, D., Pancheva, R., Fiore, M., & Tarani, L. (2025). Water-Soluble Vitamins: Hypo- and Hypervitaminosis in Pediatric Population. Pharmaceutics, 17(1), 118. https://doi.org/10.3390/pharmaceutics17010118

**The discussion is comprehensive and effectively interprets the results in the context of existing literature. It highlights the potential mechanisms by which P. dioscoridis exerts its therapeutic effects, particularly in relation to dopamine modulation and hormonal balance. The discussion also appropriately addresses the limitations of the study, such as the need for further clinical validation.**

Author response: we would like to thank the reviewer for this detailed encourging comments.

**However, the section could be more focused in some areas, particularly in the discussion of the chemical components of P. dioscoridis. While the identification of flavonoids and phenolic acids is mentioned, the discussion could delve deeper into how these specific compounds might interact with biological pathways relevant to PCOS.**

Author response: we added possible biological pathways.

Metformin improves androgen, estrogen, and progesterone levels in this study, however, P. dioscoridis extract gives more significant results. The effect of metformin may be attributed to its insulin sensitivity improvement in PCOS [69]. On the other hand, this search demonstrated the therapeutic potential of P. dioscoridis on hormonal disturbance in letrozole-induced PCOS rats. Despite that the pathogenesis of PCOS is still limited, Yuan et al., 2025 demonstrated a link between PCOS pathogenesis and hormonal fluctuations in the levels of LH, FSH, androgens, progesterone, and estrogen [70]. Flavonoids, being one of the components of P. dioscoridis have been shown to lower LH and testosterone levels in PCOS rat models through modulating the IL-6-mediated JAK2\STAT3 signaling pathway [71]. Quercetin, a flavonoid found in P. dioscoridis extract, increases CYP19A1 and CYP11A1 expressions leading to elevating estrogen levels in letrazole-induced PCOS mice model [72], in addition to improving insulin resistance through its antioxidant properties [73]. Caffeic acid a phenolic acid derivative of P. dioscoridis, alleviates endoplasmic reticulum stress and inhibits 3β-HSD protein in DHEA-induced PCOS rat model [74]. All these multi-target properties in P. dioscoridis extract give it the privilege over metformin in the treatment of this heterogenous disease.

Supporting reference:

[69]	R. Carvajal et al., “Metformin augments the levels of molecules that regulate the expression of the insulin-dependent glucose transporter GLUT4 in the endometria of hyperinsulinemic PCOS patients,” Hum. Reprod. Oxf. Engl., vol. 28, no. 8, pp. 2235–2244, Aug. 2013, doi: 10.1093/humrep/det116.

[70]	J. Yuan, Z. Li, Y. Yu, X. Wang, and Y. Zhao, “Natural compounds in the management of polycystic ovary syndrome: a comprehensive review of hormonal regulation and therapeutic potential,” Front. Nutr., vol. 12, p. 1520695, Feb. 2025, doi: 10.3389/fnut.2025.1520695.

[71]	Y. Zhou, L. Lv, Q. Liu, and J. Song, “Total flavonoids extracted from Nervilia Fordii function in polycystic ovary syndrome through IL-6 mediated JAK2/STAT3 signaling pathway,” Biosci. Rep., vol. 39, no. 1, p. BSR20181380, Jan. 2019, doi: 10.1042/BSR20181380.

[72]	M. Z. U. H. Shah et al., “Effect of quercetin on steroidogenesis and folliculogenesis in ovary of mice with experimentally-induced polycystic ovarian syndrome,” Front. Endocrinol., vol. 14, p. 1153289, 2023, doi: 10.3389/fendo.2023.1153289.

[73]	F. Moradi Negahdari, M.-A.-R. Hadjzadeh, Z. Gholamnezhad, F. Sohrabi, and Z. Samadi Noshahr, “The Protective Effects of Trans-Anethole against Polycystic Ovary Syndrome Induced Histopathological and Metabolic Changes in Rat,” Int. J. Fertil. Steril., vol. 16, no. 3, pp. 192–199, Aug. 2022, doi: 10.22074/ijfs.2021.532941.1148.

[74]	Y.-F. Chiang et al., “Caffeic acid’s role in mitigating polycystic ovary syndrome by countering apoptosis and ER stress triggered by oxidative stress,” Biomed. Pharmacother. Biomedecine Pharmacother., vol. 166, p. 115327, Oct. 2023, doi: 10.1016/j.biopha.2023.115327.

**Additionally, the discussion could benefit from a more critical evaluation of the comparative efficacy of P. dioscoridis versus metformin, particularly in light of the study's findings.**

 Author response: We totally agree with the reviewer for this important point. We added this point in the discussion. It is highlighted in yellow.

**The comparative effect of metformin and *P. dioscoridis* on hormonal modulation in PCOS**

Metformin improves androgen, estrogen, and progesterone levels in this study, however, P. dioscoridis extract gives more significant results. The effect of metformin may be attributed to its insulin sensitivity improvement in PCOS [69]. On the other hand, this search demonstrated the therapeutic potential of P. dioscoridis on hormonal disturbance in letrozole-induced PCOS rats. Despite that the pathogenesis of PCOS is still limited, Yuan et al., 2025 demonstrated a link between PCOS pathogenesis and hormonal fluctuations in the levels of LH, FSH, androgens, progesterone, and estrogen [70]. Flavonoids, being one of the components of P. dioscoridis have been shown to lower LH and testosterone levels in PCOS rat models through modulating the IL-6-mediated JAK2\STAT3 signaling pathway [71]. Quercetin, a flavonoid found in P. dioscoridis extract, increases CYP19A1 and CYP11A1 expressions leading to elevating estrogen levels in letrazole-induced PCOS mice model [72], in addition to improving insulin resistance through its antioxidant properties [73]. Caffeic acid a phenolic acid derivative of P. dioscoridis, alleviates endoplasmic reticulum stress and inhibits 3β-HSD protein in DHEA-induced PCOS rat model [74]. All these multi-target properties in P. dioscoridis extract give it the privilege over metformin in the treatment of this heterogenous disease.

The comparative effect of metformin and P. dioscoridis on ovarian morphological alterations

Histological analysis revealed that the PCOS + metformin group exhibited fewer cystic and atretic follicles and a thinner theca than the PCOS group. Similar findings have been reported in earlier research [75], [76], [77].

The potential mitigating effects of metformin and P. dioscoridis on depression and anxiety:

Metformin improved anxiety and depression-related disorder, however, its effect was less pronounced than P. dioscoridis. The therapeutic potential of metformin may be attributed to its antioxidant property since Kakhki et al, 2024 linked the antidepressive and anxiolytic properties of metformin to the attenuation of neuroinflammation and oxidative stress induced by lipopolysaccharide-induced inflammation [85]

Supporting reference:

[69]	R. Carvajal et al., “Metformin augments the levels of molecules that regulate the expression of the insulin-dependent glucose transporter GLUT4 in the endometria of hyperinsulinemic PCOS patients,” Hum. Reprod. Oxf. Engl., vol. 28, no. 8, pp. 2235–2244, Aug. 2013, doi: 10.1093/humrep/det116.

[70]	J. Yuan, Z. Li, Y. Yu, X. Wang, and Y. Zhao, “Natural compounds in the management of polycystic ovary syndrome: a comprehensive review of hormonal regulation and therapeutic potential,” Front. Nutr., vol. 12, p. 1520695, Feb. 2025, doi: 10.3389/fnut.2025.1520695.

[71]	Y. Zhou, L. Lv, Q. Liu, and J. Song, “Total flavonoids extracted from Nervilia Fordii function in polycystic ovary syndrome through IL-6 mediated JAK2/STAT3 signaling pathway,” Biosci. Rep., vol. 39, no. 1, p. BSR20181380, Jan. 2019, doi: 10.1042/BSR20181380.

[72]	M. Z. U. H. Shah et al., “Effect of quercetin on steroidogenesis and folliculogenesis in ovary of mice with experimentally-induced polycystic ovarian syndrome,” Front. Endocrinol., vol. 14, p. 1153289, 2023, doi: 10.3389/fendo.2023.1153289.

[73]	F. Moradi Negahdari, M.-A.-R. Hadjzadeh, Z. Gholamnezhad, F. Sohrabi, and Z. Samadi Noshahr, “The Protective Effects of Trans-Anethole against Polycystic Ovary Syndrome Induced Histopathological and Metabolic Changes in Rat,” Int. J. Fertil. Steril., vol. 16, no. 3, pp. 192–199, Aug. 2022, doi: 10.22074/ijfs.2021.532941.1148.

[74]	Y.-F. Chiang et al., “Caffeic acid’s role in mitigating polycystic ovary syndrome by countering apoptosis and ER stress triggered by oxidative stress,” Biomed. Pharmacother. Biomedecine Pharmacother., vol. 166, p. 115327, Oct. 2023, doi: 10.1016/j.biopha.2023.115327.

[75]	S. Furat Rencber et al., “Effect of resveratrol and metformin on ovarian reserve and ultrastructure in PCOS: an experimental study,” J. Ovarian Res., vol. 11, no. 1, p. 55, Jun. 2018, doi: 10.1186/s13048-018-0427-7.

[76]	A. A. Morsi, E. A Mersal, A. Razik H Farrag, A. M Abdelmoneim, A. M Abdelmenem, and M. S Salim, “Histomorphological Changes in a Rat Model of Polycystic Ovary Syndrome and the Contribution of Stevia Leaf Extract in Modulating the Ovarian Fibrosis, VEGF, and TGF-β Immunoexpressions: Comparison with Metformin,” Acta Histochem. Cytochem., vol. 55, no. 1, pp. 9–23, Feb. 2022, doi: 10.1267/ahc.21-00081.

[77]	H. M. Rabah *et al.*, “Novel insights into the synergistic effects of selenium nanoparticles and metformin treatment of letrozole - induced polycystic ovarian syndrome: targeting PI3K/Akt signalling pathway, redox status and mitochondrial dysfunction in ovarian tissue,” *Redox Rep. Commun. Free Radic. Res.*, vol. 28, no. 1, p. 2160569, Dec. 2023, doi: 10.1080/13510002.2022.2160569.

[85] F. S. H. Kakhki et al., “The Antidiabetic Drug Metformin Attenuated Depressive and Anxiety-like Behaviors and Oxidative Stress in the Brain in a Rodent Model of Inflammation Induced by Lipopolysaccharide in Male Rats,” Endocr. Metab. Immune Disord. Drug Targets, vol. 24, no. 13, pp. 1525–1537, 2024, doi: 10.2174/0118715303275039231228065050.
